# Gaming science: the “Gamification” of scientific thinking

**DOI:** 10.3389/fpsyg.2013.00607

**Published:** 2013-09-09

**Authors:** Bradley J. Morris, Steve Croker, Corinne Zimmerman, Devin Gill, Connie Romig

**Affiliations:** ^1^Department of Lifespan Development and Educational Science, Kent State UniversityKent, OH, USA; ^2^Department of Psychology, Illinois State UniversityNormal, IL, USA

**Keywords:** scientific reasoning, science education, cognitive development, motivation, metacognition, technology in education, video games

## Abstract

Science is critically important for advancing economics, health, and social well-being in the twenty-first century. A scientifically literate workforce is one that is well-suited to meet the challenges of an information economy. However, scientific thinking skills do not routinely develop and must be scaffolded via educational and cultural tools. In this paper we outline a rationale for why we believe that video games have the potential to be exploited for gain in science education. The premise we entertain is that several classes of video games can be viewed as a type of cultural tool that is capable of supporting three key elements of scientific literacy: content knowledge, process skills, and understanding the nature of science. We argue that there are three classes of mechanisms through which video games can support scientific thinking. First, there are a number of motivational scaffolds, such as feedback, rewards, and flow states that engage students relative to traditional cultural learning tools. Second, there are a number of cognitive scaffolds, such as simulations and embedded reasoning skills that compensate for the limitations of the individual cognitive system. Third, fully developed scientific thinking requires metacognition, and video games provide metacognitive scaffolding in the form of constrained learning and identity adoption. We conclude by outlining a series of recommendations for integrating games and game elements in science education and provide suggestions for evaluating their effectiveness.

## Introduction

### Science education and its role in the twenty-first century economy

*Scientific literacy* describes the skills that are required by citizens in a scientifically advanced democracy. We propose that students, citizens, and politicians need to understand how to investigate, evaluate, and comprehend science content (e.g., climate change, evolution, vaccinations), processes (e.g., how to test hypotheses effectively), and products (e.g., evaluating data about the most effective cancer treatments), as well as possess positive attitudes toward science (e.g., the usefulness of data when evaluating policy). The authors of a National Research Council (National Research Council, 2010) report argued that science is *the* discipline that should convey those skills required for a twenty-first century workforce, such as non-routine problem solving, adaptability, complex communication/social skills, self-management, and systems thinking. Creating a scientifically literate population requires strong science education. In this paper outline a rationale for why we believe that video games have the potential to be exploited for gain in science education.

Science operates and develops at multiple spatiotemporal scales; it is simultaneously an individual and social activity that uses and creates cultural tools. We use the phrase *cultural tools* following Vygotsky (1986) to describe tools such as language, cognition, and information seeking strategies that augment human cognition and are used in both formal (e.g., classroom instruction) and informal education (e.g., parent child interactions; Rogoff, 1990). Cultural tools can be conceptual (e.g., instruction in critical thinking) or concrete (e.g., notebooks, scientific instruments). As developmental psychologists, we are interested in the factors that influence the origins and growth of scientific thinking across the lifespan, from the child in a science classroom to the scientifically literate adult or practicing scientist. As is the case with psychological studies of the basic cognitive mechanisms involved in reading and mathematical thinking, basic research on scientific thinking can and should inform educational practice.

### Games as cultural tools in science education

The key to creating a scientifically literate workforce is to make changes to science education (National Research Council, 2010). We suggest that one way to engineer modern science education to be able to fill the needs of a twenty-first century citizenry and workforce is to *game* the education system by incorporating the lessons that we have learned about the effectiveness of video games to produce behavioral and cognitive change (McGonigal, 2011). Specifically, we suggest that science education can be improved by incorporating key features of games that influence motivation, cognition, and metacognition. Games may serve as a useful cultural tool through which instruction can effectively make use of existing capacities (Greenfield, 1994). Rather than thinking of video games as the next educational panacea, we need to consider how games might promote effective science education by analyzing game elements and their relation to developmental mechanisms. Promoting optimal health is a useful analogy: optimal health is the result of many contributing factors, rather than a single, causal factor. For example, eating healthy foods, making healthy choices (e.g., not smoking cigarettes), effectively managing stress, having a supportive social network, and regular exercise all contribute to good health. Although each contributes, none on their own guarantees optimal health in the absence of the others. There is no “magic bullet” related to optimal health and there is no “magic bullet” in education. As in health, knowledge of the constituent components that contribute to effective education allows us to create contexts in which student learning is more likely. One of the components that we feel has the potential to contribute to modern education is the “gamification” of particular elements of education.

There is much evidence for the effects that video games—specifically action games—can have in several general cognitive domains (Bavelier et al., 2012). For example, such games have been demonstrated to enhance the spatial resolution of vision (Green and Bavelier, 2007), visual short-term memory (Boot et al., 2008), spatial cognition (Feng et al., 2007), probabilistic inference (Green et al., 2010), and reaction time (Dye et al., 2009). Although there have been suggestions that video games can improve science education, to date, the evidence has been mixed.

### The gamification of science education

McGonigal (2011) argues persuasively that it is time for us to reconsider the negative connotations that we associate with video games—that they are “escapist” or “time wasters.” McGonigal concisely defines a game with a quote from Bernard Suits (1978): “Playing a game is the voluntary attempt to overcome unnecessary obstacles” (p. 41). The key features are goals, rules, a feedback system, and voluntary participation. When the National Research Council (2011) examined the educational potential of video games, their definition included these ideas and an acknowledgement that games could include elements of fun and enjoyment, as well as strategies for controlling the game environment.

*Gamification* is a term used to describe using game elements in other environments to enhance user experience (Kapp, 2012). In this paper, our goal is to analyze the idea of the gamification of science education, by drawing on research results from cognitive and developmental psychology, and educational research to provide guidance for using existing games and for developing new games to facilitate scientific thinking skills across the science curriculum. A small number of schools in the US (e.g., the Quest2Learn schools in New York and Chicago) have begun to experiment with gamification across the curriculum, though as of yet, there are no data to evaluate its efficacy.

We assert that the development and practice of scientific thinking skills takes place in the presence of cultural tools. These tools are traditionally taken to include language, artifacts (e.g., books), and institutions (e.g., public schools; Rogoff, 1990; Lemke, 2001). However, video games and computer simulations are also examples of cultural tools that could be exploited by educators. Rather than limit these tools to create positive user experiences, their motivational and learning potential can be repurposed to enhance science education. Stated differently, we can ask: what happens when we conceptualize video games as a tool that can be used in our educational arsenal, along with paper, pencils, books, and computers?

Video games are not just a vehicle for conveying content. McClarty et al. (2012) note that games are inherently ongoing assessments. A player's abilities or knowledge of the game are constantly assessed; if the player does not perform well-enough in the game, she fails. This is because games are essentially a demonstration of a player's skills. This form of assessment is, of course, different from traditional educational assessments. Games provide an authentic context in which players can demonstrate what they have learned, as opposed to standardized tests.

### The plan for our essay

In this article, we begin with a brief review of how cognitive developmental researchers define and study scientific thinking. Longer reviews of this literature are available elsewhere (e.g., Zimmerman, 2000, 2007). Thus, our goal is to provide sufficient background to consider claims that video games may facilitate scientific thinking (e.g., Barab and Dede, 2007; Steinkuehler and Duncan, 2008). Next, we highlight the elements of science education that could be supported in gaming environments. Broadly, we consider the content, process, and nature of science. We then turn our attention to the ways in which video games may be used as educational tools. Video games are designed to keep players engaged. Games promote behavioral persistence, extended time-on-task, leveling up, and mastery approaches. They also may suppress fear of failure. Game play engagement is consistent with various theories of motivation (Ryan and Deci, 2000), positive psychology (e.g., flow; Csikszentmihalyi and Csikszentmihalyi, 1975; Csikszentmihalyi, 1990), and with educational research and theory, such as the benefits of self-directed, collaborative, and participatory learning (e.g., O'Loughlin, 1992; Gauvain, 2001). We focus on three types of scaffolds: (a) motivational scaffolds, such as feedback, rewards, and flow states that engage students relative to traditional cultural learning tools; (b) cognitive scaffolds that compensate for the limitations of the individual cognitive system, such as cognitive simulations and embedded reasoning skills; and (c) metacognitive scaffolding in the form of constrained learning and identity adoption.

In the final section, we review how these scaffolds are instantiated in gaming contexts created for entertainment and those created for instruction. Finally, we review the current evidence for games in science education and outline recommendations for how to use games and gaming elements to improve science education, and how to measure the effectiveness of gamified science instruction.

## What is scientific thinking?

Scientific thinking emerges as a product of internal (e.g., motivational, cognitive, and metacognitive components) and contextual factors (e.g., education) and functions as a specific type of information seeking (Kuhn, 2011; Morris et al., 2012). *Scientific thinking* encompasses the set of reasoning and problem-solving skills involved in generating, testing, and revising hypotheses or theories. The ability to reflect metacognitively on the process of knowledge acquisition and change is a hallmark of fully developed scientific thinking (Kuhn, 2005). As is the case with other academic skills such as reading and mathematical thinking, scientific thinking is highly educationally mediated. Unlike other basic cognitive skills (e.g., attention, perception, memory), scientific thinking does not “routinely develop,” (Kuhn and Franklin, 2006, p. 974); that is, scientific thinking does not emerge independently (i.e., ontogenetically) of science education and the cultural tools of science (e.g., mathematical tools for data analysis). Furthermore, even among scientifically educated children, adolescents, and adults, interpretation of evidence is often subject to many biases, such as the influence of prior beliefs (Kuhn and Franklin, 2006; Zimmerman and Croker, 2013).

At the individual level of analysis, scientific thinking involves the coordination of various cognitive and metacognitive skills. We can situate the basic cognitive skills within the framework proposed by Klahr and Dunbar (1988). The *Scientific Discovery as Dual Search* model (SDDS) involves coordinated search through problem spaces (i.e., a space of hypotheses and a space of experiments). Kuhn's (2005) work on scientific thinking stresses the importance of metacognitive and metastrategic skills as part of fully developed scientific thinking. In particular, she focuses on the ability to differentiate evidence and theory as distinct epistemological categories. That is, we must be able to reflect, metacognitively, on the difference between information that represents evidence from information that represents theory (or explanation for a pattern of evidence) without conflating them. A more comprehensive account situates the individual investigation, evidence evaluation, and inference skills that constitute scientific thinking within a learning environment that includes direct and scaffolded instruction, and in the support of scientific activity through the use of cultural tools (e.g., literacy, numeracy, technology; Morris et al., 2012). The history of science illustrates how highly dependent the scientific endeavor is on cultural tools and instruments (e.g., microscopes, telescopes, marine chronometers, the printing press, computers). Although educators can use these tools to teach science, they are not synonymous with science education. Cultural tools can be used in the absence of education (e.g., by practising scientists), and many of the tools that support scientific activity are not specific to science (e.g., literacy, numeracy). Because scientific reasoning does not spontaneously develop, achieving short- and long-term goals is dependent on being motivated to learn about science. Accordingly, motivation is the critical link across both short- and long-term time scales as students modify their basic cognitive skills, engage in metacognitive reflection, and acquire cultural tools. Effective science education requires the integration of these three factors. Games provide a potentially valuable tool because they provide opportunities for cognitive and metacognitive engagement and are typically highly motivating (Deater-Deckard et al., 2013).

## What elements of science education can be supported by video games?

Psychologists and educators interested in how people learn science make a distinction between *conceptual knowledge* and *science process skills*. This distinction is mirrored in the way science is taught and reflected in the National Research Council's (2012) framework for science education, which lays out a series of standards for K-12 science education. The science education standards are discussed within a framework with three broad dimensions: (a) scientific and engineering practices, (b) crosscutting concepts, and (c) core ideas. The scientific and engineering practices dimension includes asking questions, defining problems, developing and using models, carrying out investigations, interpreting evidence, constructing explanations, and designing solutions. The crosscutting concepts dimension includes understanding patterns, cause and effect, and systems and system models. The core ideas dimension includes items related to the physical sciences (e.g., matter, energy), life sciences (e.g., ecosystems, evolution), Earth and space sciences (e.g., Earth's systems, Earth and human activity), and applications of science (e.g., links among engineering, technology, science, and society). There is thus a distinction between skills and practices and content knowledge. We also focus on a third category that subsumes several ideas, largely defined as “the nature of science.” For example, some of the science learning goals that have been identified as supported in informal learning environments (National Research Council, 2009) include understanding that science is a “way of knowing.” Additionally, the ideas of scientific discourse, and self-identification as someone who knows about and uses science are seen as important components of understanding the broader institute of science situated within culture (National Research Council, 2009).

At the heart of the National Research Council's (2012) framework for science education is a conceptualization of students as scientifically literate citizens, as consumers of scientific information, and (for some students) the future producers of such information. To this end, the NRC argues that we move away from an emphasis on learning a broad array of facts and toward giving students authentic experiences with *doing* science. The NRC focus on a small set of disciplinary core ideas in engineering and physical life and earth sciences, and propose that these ideas should be taught and learned within contexts of scientific and engineering practice. Importantly, an appreciation of scientific and engineering practices should involve an understanding of how these practices as are embedded within social and cultural contexts. That is, students must recognize and appreciate that all of the concepts and procedures that we call “science” are the product of collaborative human activity: our collective, ongoing, and cumulative knowledge is produced by many scientists, many of whom work in teams, building on previous knowledge, and using cultural tools. Although some scientific research is shaped by the goals of the individuals conducting the research, much research, as well as scientists' personal goals, is driven by societal needs.

Much has been written on the rationale for including video games in educational contexts in general, and in science education in particular (e.g., Annetta, 2008; Barab et al., 2009; Mayo, 2009; National Research Council, 2011). We agree that video games can be used to scaffold internal factors, such as motivation, cognitive skills, and metacognitive skills, while also providing constrained and directed use of cultural tools, such as recording prior behaviors and outcomes, and providing task-relevant knowledge. Gee (2008a) argues that good video games mirror a formal description of how scientists approach problems: they construct a hypothesis, design an experiment to test the hypothesis, evaluate the results, and refine the hypothesis accordingly. This description of scientific behavior bears a close correspondence to Klahr's (1996, 2000; Klahr and Dunbar, 1988; Dunbar and Klahr, 1989) conceptualization of science as a search through problem spaces.

There are three different ways in which video games may support the development of scientific thinking and science education. First, there are some games, often referred to as serious educational games (Annetta, 2008), in which scientific domain knowledge is taught by using the gaming context to promote inquiry-based learning. For example, *Supercharged!* (Squire et al., 2004) is a game designed to teach principles of electromagnetism. Cheng and Annetta (2012) used a video game to give students instruction on the effects of methamphetamine on the brain, and *Immune Attack* teaches immunology concepts. These games incorporate core disciplinary ideas relating to the third dimension (i.e., *core ideas*) of the Framework for Science Education (National Research Council, 2012). Learning the wide range of discipline-specific content may be difficult to adapt to game play, given the vast number of possible science concepts that can be taught. Prensky (2011) notes that “building a game for every topic is probably not necessary” (p. 268). However, big picture “crosscutting” concepts such as systems, patterns, and causality are perfect for games and simulation.

Second, there are games in which instruction in scientific process skills is embedded. For example, *River City* is a multi-user virtual environment in which small teams of students conduct scientific investigations into an epidemic affecting a historical town (Galas and Ketelhut, 2006; Nelson et al., 2007). *Mad City Mystery* is a game designed to teach students inquiry and argumentation skills as they investigate a mysterious death (Squire and Jan, 2007). These games relate to the scientific practices in the first dimension of the National Research Council (2012) Framework.

Third, there are games that may promote the development of skills, attitudes, and values that are useful for scientific thinking or practice, but without any explicit instruction in scientific knowledge or skills. Some of these games may embed scientific practices from the first dimension of the framework, or they may support crosscutting concepts from the second dimension of the framework (National Research Council, 2012). They may also support concepts related to science as a multi-scale, social, collaborative endeavor. For example, situating cognition in a contextualized virtual environment, providing collaborative gameplay structures, and role-playing characters are elements found in many successful commercial games that could be exploited to situate gamers in the context of scientific investigation. Additionally, there are games that may not bear any obvious relationship to any of the items in the Framework, but may still promote skills useful for components of science (e.g., cognitive). For example, games that exercise spatial cognition, whether puzzle games (e.g., *Tetris*) or action games (e.g., *Medal of Honor*), may have an effect on visualization skills used in thinking about scientific concepts and processes (Feng et al., 2007; Newcombe, 2010; Newcombe and Stieff, 2012; Uttal et al., 2013). Games may also improve working memory capacity, an important element in problem solving (Hawes et al., 2013). Adachi and Willoughby (2013) report that playing strategic (as opposed to action) video games predicts higher self-reported problem solving skills. Scientific literacy skills may also be fostered in non-academic game contexts. For example, evidence of scientific discourse, model-based reasoning, and understanding of theory and evidence has been found in an analysis of *World of Warcraft* discussion board postings (Steinkuehler and Chmiel, 2006).

## How do (could) games facilitate scientific thinking?

According to a report by the Federation of American Scientists (2006), many of the features used in high-quality learning environments are also found in video games. Both well-structured classroom lessons and video games have (a) clear learning goals, (b) opportunities for practice and reinforcing expertise, (c) monitoring of progress, and (d) adaptation to the level of mastery of the learner. As in good educational experiences, video games can encourage inquiry, engage learners so that they are motivated to spend time on the task and to develop expertise, and provide a contextual bridge between the concepts learned and their applications. Video games can also scaffold the learner's development, help the learner to adapt to different levels of knowledge and motivation, and provide “infinite” patience for learners who need to attempt tasks multiple times before developing competence.

Science is concerned with causal *mechanisms* and explanation (Koslowski, 1996). To support our assertion that video games are a cultural tool that can be used to promote and encourage scientific thinking, it is necessary to outline what sort of causal powers they have to bring about these (desired) effects. In this section, we focus on ways that video games may be effective in supporting scientific thinking with respect to scaffolds in three broad domains: (a) motivation, (b) cognition, and (c) metacognition.

### Motivational scaffolds

One of the key features of video games that educators would love to exploit is their ability to motivate. Games seem to motivate people in ways that other activities often do not. For example, students often continue playing video games despite encountering frequent obstacles, yet these same students may not demonstrate the same level of persistence given setbacks in schoolwork. Motivation refers to the level of engagement related to achieving a goal and includes factors such as persistence in the face of setbacks (e.g., Henderlong and Lepper, 2002; Dweck, 2006). In order to gamify science education, it is helpful to determine why games are highly motivating.

#### Curiosity

Curiosity is the “threshold of desired uncertainty in the environment that leads to exploratory behavior” (Jirout and Klahr, 2012, p. 125). This definition suggests that an important element of motivation is the desire to bridge a gap in information between what is known and what is unknown, but which is also achievable. Curiosity is critically important in scientific thinking as it drives children's exploratory behavior when they are interested and when the task or phenomenon is not too complex (Loewenstein, 1994). There is a “sweet spot” when it comes to curiosity—an optimal level—that prompts behavior.

In games and in education, the “skill gap” or “knowledge gap” is a moving target. That is, the gap is dynamic because knowledge and experience change in real time. Maintaining an optimal skill gap is difficult in educational settings because it requires monitoring and modifying the information gap, which changes over time and differs across individuals (Singer et al., 2000). In contrast, game designers have built in the mechanism for maintaining the skill gap: *leveling*. Most games begin with simpler levels and become more complex with play. Players maintain the skill gap by moving through the game at their own pace. Additionally, players can re-play levels to practice skills before moving on to more difficult levels. Self-pacing and monitoring allow the player to monitor and modify their own skill gap.

Finally, games provide curiosity-promoting contexts in that they have optimal levels of uncertainty. One context is the world in which the player must discover the rules for action. *Portal* is a game in which players have to solve a series of puzzles by creating portals through which the character and objects teleport. Other games promote curiosity by immersing players in a virtual world. *Bioshock* is an underwater world that the player must navigate in order to survive and may prompt curiosity about the world itself and the capacities/limitations of the character being played.

#### Feedback

Feedback is an internal or external evaluation of current performance relative to a goal that allows a student to optimize performance (Powers, 1973). Feedback is clearly important in learning situations. Educational assessment is a type of feedback, and in the case of formative assessment, the idea is to provide explicit, concrete information to meet a specified criterion (e.g., demonstrating knowledge of multiplication facts).

Feedback is more effective when it provides *sufficient* and *specific* information for goal achievement and is presented relatively *close in time* to the event being evaluated (Graesser and Person, 1994; Prensky, 2001). Verbal or written feedback employed as an immediate and direct response to student academic performance has been demonstrated to be a powerful classroom intervention (Wilbert et al., 2010). Feedback can reference individual learning progress, can make social comparisons, or can refer to task criteria (Wilbert et al., 2010). Feedback occurs at varying grain-sizes from simple (e.g., information about correct/incorrect performance) to complex (e.g., extensive suggestions for revising a paper), can be positive or negative (e.g., rewards, punishments; Brophy, 1981), and may provide information about causal explanatory links (e.g., attributions for success or failure; Weiner, 1995).

Like experienced tutors, games provide *immediate* feedback at *multiple grain sizes* (Mayo, 2009). For example, in *Angry Birds*, players immediately see the impact of different birds on specific materials (e.g., wood, glass) and situations (e.g., no gravity) as well as seeing their progress in completing a level, which is related to overall difficulty. Compared to those given direct, classroom-based instruction in which students were given infrequent, general feedback (e.g., letter grades), high school students learned more about computer science concepts when these concepts were presented in a game system that provided immediate, specific feedback on performance (Papastergiou, 2009). The most effective feedback focuses on the task at hand rather than characteristics or traits, on that which is within the recipient's control, and requires more work from the recipient than from the giver (Powers, 1973).

#### Praise

Praise is a positive evaluation of performance (Henderlong and Lepper, 2002). Praise differs from feedback in that it generates self-attention by comparing performance to a standard (thus increasing intrinsic motivation; Baumeister et al., 1990). Praise also differs from rewards in that rewards are consequences that increase the frequency of a behavior (Deci et al., 1999). Praise is most effective when it is perceived as informational (rather than controlling) and when focused on processes (e.g., effort) rather than on personal traits (e.g., ability; Corpus et al., 2006). Praise influences motivation orientations by creating expectations about the extent to which performance is changeable (e.g., whether one has control over outcomes; Cimpian, 2010). More specifically, praise directed toward effort (e.g., *you worked hard*) suggests that one's effort caused success/failure and that this is a malleable, controllable factor. Conversely, praise directed toward traits (e.g., *you are smart*) suggests that success/failure was caused by the possession of a stable trait that is not controllable or malleable (Kamins and Dweck, 1999; Cimpian et al., 2007; Zentall and Morris, 2010).

Given what we know about the conditions under which praise can have the most positive effects on learning, video games can be designed to incorporate the optimal type and frequency of praise, thereby increasing motivation and persistence. By focusing praise on effort, video games often signal the need for additional effort (i.e., persistence). *Dance Dance Revolution* provides verbal praise to players during gameplay (Perfect!) and *Rock Band* praises (cheers) and criticizes (boos) players during gameplay. Multiplayer games (e.g., *Halo*) allow the possibility of praise both from the game itself and from other gamers.

#### Motivation orientations

Children's explanations for the causes of success and failure directly influence their motivation. Children with a mastery orientation believe that effort is controllable and related to success and have task mastery as their goal (Kamins and Dweck, 1999; Cimpian et al., 2007). In contrast, children with helpless orientations believe that non-controllable factors like traits (e.g., intelligence) are related to success and have performance goals (e.g., achieving external markers of validation such as awards; Kamins and Dweck, 1999). Praise for effort is linked to promoting mastery orientations, while praise for traits is linked to promoting helpless orientations (Kamins and Dweck, 1999; Cimpian et al., 2007; Zentall and Morris, 2010). Games often implicitly and explicitly promote mastery orientations (Klimmt and Hartmann, 2006). For example, the immediate feedback between player and outcome highlights the relation between effort and competence, a critical factor in increasing intrinsic motivation (Przybylski et al., 2010). Games provide a context in which players perceive high levels of control and competence related to their own actions (Ryan et al., 2006). First- and second-grade children increased the amount time-on-task for math and reading comprehension activities, compared to traditional materials, when these activities were presented in a game format (Rosas et al., 2003). Games sometimes provide explicit praise for effort that promotes mastery orientations. For example, when completing a level on *Cut the Rope*, players are given praise (e.g., Excellent) that is directed toward outcome rather than player characteristics.

#### Fun failure

In many contexts, failure and error provoke high levels of anxiety, which typically reduces motivation (Cimpian, 2010; Zentall and Morris, 2012). Anxiety is related to the consequences of failure, specifically, failure (or error) provokes anxiety in those with helpless orientations because it is a negative external consequence (Cimpian, 2010). Because error is threatening to one's self-image, praise directed to traits appears to increase threat vigilance, defined as increased attention to threatening information such as errors (Vuilleumier, 2005). Young children who received trait-based praise produced more visual fixations to errors than children who received effort-based praise (Zentall and Morris, 2012). Teachers experience anxiety related to science instruction (Cox and Carpenter, 1989) and students who experience anxiety related to science instruction often avoid science courses (Tilgner, 1990) and select non-science majors (Udo et al., 2004). In a related subject, mathematics, female teacher anxiety is related to entrenching gender stereotypes about mathematical abilities and increasing math-related anxiety in the girls in their classes (Beilock et al., 2010).

There is some evidence that error and failure may be viewed differently within gaming contexts (McGonigal, 2011). Specifically, errors in gaming contexts may not provoke anxiety and threat vigilance in the same way that it does in academic contexts. Because games often provide frequent impasses, the net impact of a single impasse is likely to be lower than in other contexts. In fact, errors within games are often viewed more as *feedback* than as external evaluation. For example, gamers playing *Monkey Bowling 2* showed physiological indications of positive affect, rather than negative affect, following errors in game play (Ravaja et al., 2005). This finding suggests that one potentially useful feature of gaming in education is changing how students interpret error, specifically, changing children's attributions of error and failure from negative external evaluation to constructive feedback.

#### Flow states

Flow has been described as an optimal state of being in which one experiences intense focus or concentration, a merging of action and awareness, and a high sense of agency (i.e., high sense of control; Nakamura and Csikszentmihalyi (2002). Flow is an important dimension of the positive psychology of gaming in that it includes positive emotions, feedback indicating how well the individual is performing the particular activity, performance that often occurs at a level above the previous skill level of the individual, and is associated with intrinsic rewards (Nakamura and Csikszentmihalyi, 2002; McGonigal, 2011). The flow state appears to combine low levels of anxiety and an optimal skill gap (Berlyne, 1960). The balance of achieving a flow state is dependent upon both the challenge and skills required for the activity (Cowley et al., 2008). If the challenge is too difficult, the player could experience anxiety. If the challenge is too easy, then boredom could occur.

Because the components necessary to elicit flow discussed above are often present within video games (e.g., matching skill level to task difficulty) games are seen as *flow machines* (McGonigal, 2011). Playing a video game provides immediate feedback for contextual learning and matches player skills to game difficulty. Games often elicit intense focus of attention associated with flow states (Przybylski et al., 2010).

Although games appear to be well-suited to eliciting flow states, is eliciting flow useful for science education? Increasing the positive emotional experience associated with science education is a potentially important factor in engagement. Certainly, it would be helpful for students to be as engaged in activities related to science education as they are engaged in a video game such as *World of Warcraft* for the simple reason that time spent in deliberate practice is highly related to emerging expertise (Son and Simon, 2012). Gamification can be useful in increasing high levels of sustained attention, which are critical in the type of deliberate practice associated with emerging expertise. Flow also offers the ability to overcome temporal discounting associated with achieving long-term rewards with both less time and energy spent attaining that reward than traditional education approaches.

Instructional methods such as direct instruction (e.g., Klahr and Chen, 2011) and discovery learning (e.g., Schwartz et al., 2011) may differ in the extent to which they elicit flow. For example, discovery learning may be better suited for eliciting flow than direct instruction because discovery learning is self-paced, provides more immediate feedback/rewards, exploration may provide a means for regulating the information gap, and the stakes of error/failure may be lower compared to direct instruction. Although discovery learning has some advantages, students often fail to learn during unstructured discovery learning (i.e., providing little or no guidance to students; Klahr, 2012).

One open question is the extent to which eliciting flow states would demonstrate benefits in science education outcomes. Because one must learn to progress within a game, learning is inherent in game play. However, learning appears to be a condition upon which flow states occur. Achieving the types of skills most associated with flow states (e.g., as seen in experts; Bakker, 2008) requires deliberate practice that is associated with few immediate rewards. For example, expert pianists were more likely to experience flow states as the amount of rehearsal increased (de Manzano et al., 2010). Students competing in U.S. national spelling bees were more likely to be motivated to persist in deliberate practice even when they were showing few signs of improvement (Duckworth et al., 2011). This level of persistence was associated with extremely high task performance and suggests that motivation for practice, even when associated with immediate rewards, is linked to mastery. The metacognitive reflection necessary for increasing science understanding may also be at odds with flow states. If flow is more likely to occur when implementing sequences of rehearsed procedures, then the deliberative and metacognitive processes of scientific reasoning may be a poor fit for gaming elements.

Perhaps a better fit between flow and science education is to determine the contexts, tasks, and skill levels in which eliciting flow would be useful for developing science knowledge and skills. For example, parts of skill learning and active investigation are likely to be enhanced by the positive experiences associated with achieving flow, similar to videogames. These tasks will be most useful if they are paired with tasks that promote deliberative, goal-directed effort, and metacognitive reflection about the processes and content under study.

### Cognitive scaffolds

The cognitive skills used in scientific thinking include identifying problems, generating hypotheses, designing experiments, collecting data, evaluating evidence, and making inferences (Zimmerman, 2007). In authentic science and in all high-level cognitive work, when we solve complex problems, we use tools in the environment to reduce cognitive load. We transform complex serial problems into the type of pattern-recognition-based problems we are good at (Mareschal et al., 2007). In science, our toolkit contains both *conceptual tools*, such as hypothesis testing and evidence evaluation, and *concrete tools*, such as notebooks, diagrams, and computers (Morris et al., 2012; Zimmerman and Croker, in press). Over the last decade, many educational games have been developed with a consideration of the conceptual tools (i.e., cognitive skills) used in scientific thinking. In the following section, we review five ways in which games scaffold cognitive processes: providing simulations, situating cognition, distributing knowledge and promoting collaboration, promoting the values of science, and engaging in real-world problem solving.

#### Simulation

Many educational video games are explicitly designed so that players enter physical, biological, or social systems. For example, *River City* is a multi-user virtual environment in which small teams of students conduct scientific investigations into an epidemic affecting a historical town (Galas and Ketelhut, 2006; Nelson et al., 2007). *River City* was designed to help low-achieving students improve in science, specifically with respect to knowledge of biology and ecology, hypothesis generation, and experimental design (Dede et al., 2005). The game is inquiry-based: students have to identify problems, collect and evaluate data, and draw conclusions. Middle school students who played this game outperformed a control group with respect to their knowledge of biology. Classrooms using *River City* had higher levels of engagement in science, better attendance, and fewer disruptions (Dede et al., 2005).

In *Supercharged!*, students inhabit a physical system. Players pilot a spaceship that can adopt properties of a charged particle. In the game, students navigate through a high school electrostatics curriculum. Squire et al. (2004) found that students who played *Supercharged!* developed a deeper understanding of physics, developed a better understanding of the representations used in physics textbooks than students who did not play the game, and that lower-achieving students experienced the greatest gains in understanding.

In *Quest Atlantis* (Barab et al., 2005), elementary and middle schoolers learn elements of the science curriculum related to the social aspects of science such as social responsibility and environmental awareness. The skills promoted in *Quest Atlantis* are not just those needed by scientists, but by scientifically literate citizens. When playing this game, children enter a world in which pro-science values are fostered, a point we shall return to shortly.

*Environmental Detectives* (Klopfer et al., 2004; Klopfer and Squire, 2008) blurs the border between simulation and reality by combining a video game played on a portable computer with navigation through real-world spaces. The objective is to teach scientific and engineering skills through action, identity, and collaboration. In *Environmental Detectives*, players work in teams to research a chemical spill on a college campus. The game features many aspects of scientific thinking, including data collection, data evaluation, asking questions, discussion, and argumentation in teams, and understanding what knowledge is needed to proceed. This game is designed to be authentic with respect to the practices of environmental engineers, involving the integration of primary and secondary data. The problem presented to players is complex, ambiguous, and open-ended, unlike typical school science assignments.

*Biohazard* is an undergraduate-level biology and environmental science game that was later used to help first responders learn how to deal with chemical attacks in public spaces. Players work in teams to save civilians in the event of gas attack, and to locate the source of the gas. *Biohazard* embeds learning scientific knowledge about chemicals and the symptoms of exposure alongside investigation skills and increased communication and problem solving skills among players (Squire, 2003; cited in Squire and Jenkins, 2003).

The games discussed in this section were designed to teach scientific and investigative practices using conceptual tools. However, there is a much wider class of video game that aids problem solving through concrete tools, and gives us practice in using such tools. For example, many strategy games (e.g., *Age of Empires*) require extensive use of maps. Thus, the possible utility of video games as a vehicle for the development of scientific thinking goes beyond the explicit inclusion of cognitive skills relating to generating, testing, and revising hypotheses. The educational games described above involve simulations, but simulations are also a feature of commercial games. Gee (2009) argues that commercial video games are ideal tools for learning for several reasons; one of which is that they are simulations. Scientists use simulations to make predictions, manipulate variables, observe effects, and try to understand complex systems. Although the scientist is outside the system, she may often try to understand it by situating herself inside the system. Scientists can imagine they are in their simulations; they can talk about how they might behave if they were an element in a complex system, such as an electron or an ant. Video games explicitly place the player inside simulations in which variables interact. Games are set up as problems, with goals, operations, strategies, and resources. Players must learn how the system works: what the rules are, what works, and what does not work. Scientists do something very similar in that they try to understand “rules” underlying systems. Video games are thus inherently about induction and rule discovery, regardless of the content of the game (Greenfield et al., 1994). Furthermore, video games foster an attitude or stance toward simulations similar to that adopted by people doing authentic science. The fact that games are, in this way, similar to what scientists do does not mean that players learn scientific concepts, but rather that such games lead people toward systems thinking.

#### Situating cognition

A second important way in which video games—whether educational or commercial—support cognitive skills is through situating cognition. In video games, cognition is “situated” because games provide contextualized opportunities for the application of knowledge and the exercise of skills and strategies. Gee (2008b) refers to games as “action- and goal-directed simulations of embodied experience” (p. 254). In accord with ecological, situated, and embodied approaches to cognitive science (e.g., Clark and Chalmers, 1998; Spivey, 2007), we can conceptualize thinking as being engaged in simulations preparing us for situated action. The simulations we build when we think are geared toward goals, or “winning.” Gee's argument is that simulation games are an externalized version of the mind and, as such, provide us with a support for engaging in thought. Educators and game developers have leveraged this metaphor of games-as-mind by designing games in which players act in the role of scientists. For example, in both *River City* and *Environmental Detectives*, players are given problems similar to those faced by scientists. The aim is to get players to approach these problems as a scientist would. In addition, games can use the adoption of identities to foster more meaningful learning. In science classes, students are given problems and goals that are posed by others. However, practicing scientists define their own goals, within a larger social-historical context. As a result, personal and professional identities of scientists are aligned. Students do not get this identity alignment unless they also get to set their own goals, or at least come to understand externally imposed goals in ways that are meaningful to them (Gee, 2005a).

#### Distributed knowledge (or virtual collaboration)

One way in which video games support players' adoption of goals as meaningful is through distributed knowledge and skills (Gee, 2005a, 2009). Some games involve cooperating with virtual characters who possess knowledge and skills the player lacks; essentially virtual mentors who provide scaffolding and engage in instructional dialogs with the player (Wood et al., 1976; Chi, 2009). The player and the virtual characters work as a team, each deploying specialist knowledge, in a context in which all have shared values and attitudes toward the tasks the player is engaged in. Distributing knowledge in this way reduces the cognitive load on the player. The player can then perform in the game before developing full competence. Stated differently, mastery of the game is not necessary in order to play. The notion of performance before competence is at odds with the traditional educational process of competence before performance, in which students are required to learn a lot before engaging in practice. Video games could be designed such that players act as team members engaged in scientific activities. One of the aims here would be for the player to internalize the values of scientists. Gee (2005b) refers to the distribution of knowledge and skills as *authentic professionalism*, arguing that solving specific problems as part of such a team gives players the experience of how real-life professionals solve problems, regardless of whether the domain is law, medicine, urban planning, or science. Embedding a player in a distributed knowledge domain allows the player to observe and engage in a discourse that is shaped by the values shared by experts in a given area of expertise. In order to show students the worlds of science, we need to situate them within the discourse and values of science—and games may be a way of achieving this embeddedness.

#### Values and identity

Shaffer (2005) refers to experts' communities of practice as having *epistemic frames*, or ways of knowing about the world influenced by specific disciplines. These frames are constituted by practice, identity, interest, understanding, and epistemology. When students become part of a culture of science, for example, their epistemic frames are the internalization of scientific conventions. Shaffer proposes taking epistemic frames and using them to create epistemic games. Some examples of epistemic games, in which players are embedded in a specialist community, include *Madison 2200* (urban planning), *Digital Zoo* (engineering), and *Nephrotex* (engineering). *Digital Zoo* helps students understand principles of engineering, and also the ways in which engineers think (Svarovsky, 2009). *Nephrotex* is aimed at first-year college students, who engage in engineering role play in the game in order to develop not just knowledge and skills, but also the values and identities of engineers (Shaffer et al., 2011). Arastoopour et al. (2012) report that women who played *Nephrotex* developed more positive views of engineering as a career than women who did not play the game.

#### Preparation for real-world problem solving

Video games can support training and practice in deploying cognitive skills essential for scientific thinking and also provide an apprenticeship in thinking—and acting—as a scientist. Apprenticeship in thinking and problem solving through play and other informal teaching is not a recent innovation. It is typical for children to be given learning experiences that represent the kinds of problems they will face in adult life (Rogoff, 1990; Mareschal et al., 2007). In recent history we have come to rely on education in addition to informal experiences. In schools we formalize the problems that children will need to master for survival (broadly construed), such as literacy and numeracy. Video games present opportunities for us to expose children and students to problems they will face in the future, such as the energy crisis and climate change.

These socio-scientific problems facing us are complex and large-scale and require us to think of potential solutions in terms of decades and centuries. McGonigal (2011) argues that “God games” (e.g., *Civilization*, *Black & White*) foster thinking about how events unfold over long timespans and about our actions in terms of long-term outcomes, a critical component of linking observations over time to detect patterns in evidence, both important skills in scientific reasoning. In addition, these games promote systems thinking, in which we come to conceptualize worlds in terms of interconnected parts, and understand how the impact of an intervention in one subsystem affects other systems. God games also allow players to generate and test different solutions to problems. Players can design and conduct tests, employ strategies, and run simulations to look for the strategy that yields the best outcomes.

Forecasting games also promote thinking about the future. For example, in *World Without Oil*, players are asked what would happen if oil started running out today. Players devise scenarios of what would happen, but also think about how to overcome the problems the oil shortage would create. Because the game reflects a real-world problem, players are actually thinking and planning about something that *will* occur. They cooperate and collaborate to create solutions for when it does happen. In addition to engaging people in thinking about a problem, the game also led some people to change their habits (e.g., reusing bags, driving less, recycling; McGonigal, 2011). Turning a future problem into a game allows us to take advantage of the voluntary participation component of gaming. If a problem hits us now, we are not engaging with it voluntarily, and do not spend as much time engaging in a thoughtful, motivated way. Voluntary gaming, in contrast, can foster interest, curiosity, motivation, effort, and optimism. There are no immediate, real, negative outcomes, so players have space to think about the problem.

*EVOKE* is a multiplayer game designed to promote young people's thinking about real-world problems in a game context. Examples of the problems players have engaged with include food, energy, and human rights. African universities want to engage students in real-world problems; *EVOKE* may meet this kind of need. *EVOKE* has led to real-world implementations of proposed solutions. For example, a food-production skills program in South Africa, and converting boats to solar power in Jordan to reduce fuel and the impact on nature (McGonigal, 2011).

### Metacognitive scaffolds

Metacognition is an important component of scientific thinking involving the accurate monitoring of one's own knowledge and skills. Kuhn (1989, 2005) argues that the defining feature of scientific thinking is the set of cognitive and metacognitive skills involved in differentiating and coordinating theory and evidence. Kuhn (2005) further posits that the effective coordination of theory and evidence depends on three metacognitive abilities: (a) encoding and representing evidence and theory separately, so that relations between them can be recognized; (b) treating theories as independent objects of thought (i.e., rather than a representation of “the way things are”); and (c) recognizing that theories can be false, setting aside the acceptance of a theory so evidence can be assessed to determine the veridicality of the theory.

These metacognitive abilities are necessary precursors to sophisticated scientific thinking, and represent one of the ways in which children, adults, and professional scientists differ. When we consider the larger social context, it is clear that these types of metacognitive skills that are highly valued by the scientific community may be at odds with the cultural and intuitive views of the individual reasoner (Lemke, 2001). Evidence is not just evaluated in the context of the science investigation and science classroom, but within personal and community values. In order for children's behavior to go beyond demonstrating the correctness of one's existing beliefs (e.g., Dunbar and Klahr, 1989) it is necessary for meta-level competencies to be developed and practiced (Kuhn, 2005). With metacognitive control over the processes involved, children can change what they believe based on evidence and, in doing so, become cognizant that they are changing a belief, and understand *why* they are changing that belief. Thus, sophisticated reasoning involves both the use of various strategies involved in hypothesis testing, induction, inference, and evidence evaluation, and a meta-level awareness of when, how, and why one should engage in these strategies. In the following section, we review five types of metacognitive scaffolds: knowledge, contexts, identity, memory, and strategies.

#### Metacognitive knowledge

As in science—and life in general—gamers need to metaphorically stand outside the task they are engaged in and examine their assumptions or beliefs about how a system works. In video games, players often need to ask themselves whether an aspect of the game really works the way they think it does, or whether they have all the knowledge needed to carry out a particular operation in order to reach a goal. Asking these questions represents a metacognitive awareness of knowledge. Gamers also need to engage in metacognitive regulation: planning, monitoring, and evaluating actions and outcomes. We argue that effective and engaging video games promote metacognitive activity by making it hard for players to make progress unless they reflect on how to succeed in a task and ask what knowledge or skills they are missing when they cannot overcome an obstacle. Furthermore, activities—including games—that are most effective in developing, supporting, and promoting scientific thinking *must* include metacognitive awareness of, and reflection on, knowledge and practices. Failure to make progress in educational contexts can result in disengagement from the activity, whereas failure in video games often results in persistence. Some reasons for this situation are described in the section on motivation above. In overcoming obstacles, and in preparation for repeating behaviors that are successful, it is very useful to understand why a behavior led to success, and in what contexts it should be used.

Metacognitive understanding of concepts and skills is facilitated when they are contextualized. Gee (2005a) notes that in games, unlike in education, players do not start by learning a set of isolated facts; they learn by taking on an identity and becoming immersed in a virtual world. Players learn through experience, but the experiences are constrained and scaffolded. For example, Evans and Biedler (2011) discuss “Mission: Evolution,” an informal science learning project in which middle and high school students explored concepts in evolutionary biology using *Spore Galactic Adventures*. Students would construct a game illustrating a scientific concept, such as adaptation or speciation, only to realize they did not have all the conceptual knowledge necessary to complete the task. In order to overcome the obstacle of insufficient knowledge, students had to use metacognitive skills to reflect on their knowledge and identify what they knew and what they did not know. Constrained exploration has been used as an alternative to direct instruction and shows promise in instructional contexts (Schwartz et al., 2011). Eighth graders learned and transferred knowledge about the concept of density in a highly constrained learning environment that provided contrasting examples to support reflection as they learned (Schwartz et al., 2011).

In science contexts for which students do not possess a large body of knowledge to guide their investigations, they cannot use heuristics to constrain the problem space. Thus, prior knowledge and metacognitive skills are necessary to engage in unguided scientific inquiry. Gee (2005a) gives the example of Galileo's discovery of the laws of pendulums. Galileo applied his existing knowledge of geometry to the problem. Without this knowledge, the problem space is much larger. Giving children pendulums to play with and expecting them to recreate Galileo's work is to give them a task more difficult that the one that faced Galileo. Gee argues that domain knowledge can best be described as the ability to use prior knowledge and conjecture to construct mental models or simulations to plan actions. In science, such mental activity is typically referred to as *hypothetical thinking* (Amsel, 2011). Gamers can then apply their existing knowledge to evaluate the outcome and decide whether the simulation is a good one. A verbal description of a scientific concept is less helpful than a simulation of how the concept applies in a situation. The latter, according to Gee, prepares one for action and dialog in the relevant domain. Facts are easier to learn when they are components needed to build good simulations rather than when they are not embedded in a context. Gee contends that, just as in games, students can use simulations in science domains to consider several actions and the associated outcomes, plan a course of action, evaluate the outcomes, and use this information to construct new simulations for better outcomes in the future.

#### Context

Treating theory and evidence as distinct—a key metacognitive skill in scientific thinking—requires us to suspend prior beliefs in order to evaluate evidence without prejudice. Many video games require the suspension of prior beliefs, as there may be many aspects of the virtual game world that conflict with our knowledge and experience of reality. For example, all of our assumptions about the behaviors of birds and pigs are violated in *Angry Birds*, and any game in which characters possess magical abilities, such as *World of Warcraft* or the *Final Fantasy* series, violate our assumptions about physics. In other games—and in keeping with Arthur C. Clarke (1962) famous assertion—such abilities may be cast as the result of advanced technology rather than magic (e.g., the *Portal* gun). Video games present explicit fantasy contexts. In fantasy contexts it is easier to accept evidence that contradicts prior beliefs than in the real world, particularly for young children (Amsel et al., 2005). Children also tend to treat improbable physical, biological, or psychological events as impossible situations (Shtulman and Carey, 2007; Shtulman, 2009). Gamers, in contrast, can soon come to realize and accept that the laws governing a game are not necessarily the same as those that apply in the real world. Gamers can propose hypotheses about the nature of the virtual world without conflict, whereas in science the conflict between naïve beliefs and a scientific understanding can be difficult to negotiate.

#### Identity

Another aspect of many games that reduces conflict between existing knowledge and values and scientific knowledge and values is the use of a virtual identity. In the classroom, a child has an identity that has values about and attitudes toward science. In some cases, this identity may be that of someone who is good at science and who comes from a family in which science is valued. In other cases, the child's identity may be that of someone who does not enjoy school, does not do well in science classes, who may be anxious about science and mathematics, has gender-related misconceptions about science and math abilities, or has family values that conflict with scientific explanations of the world (Lemke, 2001; Gee, 2003). In games in which players assume the role of a given or self-created character (e.g., *World of Warcraft*), they can behave as the character would behave, and adopt beliefs and values that may not be the same as their own. If this aspect of games were incorporated into science learning, children could adopt the virtual identity of a scientist; this could reduce anxiety as well as reducing the conflict between what a child may assume to be true and what the evidence suggests. The learner can adopt two perspectives simultaneously—that of herself and that of a scientist, in the same way children regularly do in a game context. There is empirical evidence that adopting a scientific perspective has an effect on college students' beliefs about science and performance on science and reasoning problems. Amsel et al. (2008) asked some participants to complete a ratio-bias task whilst adopting the perspective of a logical person and others to just complete the task. The former group gave more analytic responses than the latter. The manipulation enabled participants to inhibit heuristic responses. Amsel et al. (2009) asked introductory psychology students to think like their professor when completing a questionnaire on beliefs about psychology, which increased the number of students who endorsed psychology as a science. Similarly, when Amsel and Johnston (2008) asked physics students to think like their physics professor, the students exhibited improved performance on physics problems. Furthermore, giving children choices about the name of their character and the icon representing the player can result in increased willingness to engage in an educational game (Cordova and Lepper, 1996).

#### Metamemory

In addition to an awareness of the knowledge required to solve a problem, scientific practice is facilitated by an awareness of the limitations of our cognitive abilities, such as memory. Many studies demonstrate that both children and adults are not always metacognitively aware of their memory limitations while engaged in investigation tasks (e.g., Siegler and Liebert, 1975; Dunbar and Klahr, 1989; Gleason and Schauble, 2000; Trafton and Trickett, 2001; Garcia-Mila and Andersen, 2007). Children are less likely than adults to record experimental designs and outcomes, or to review any notes they do make, despite task demands that clearly necessitate a reliance on external memory aids. Instead, children may depend on familiarity or strength of prior beliefs (Kanari and Millar, 2004). Video games can also help to reduce the demands placed on working memory while engaged in problem-solving behaviors as many games keep track of resources and prior accomplishments. Further, if a player forgets whether she has tried to solve a problem in a particular way, she can try again with little cost. Although video games probably do not foster the development of metamemory, they can reduce cognitive load.

#### Metastrategic competence

Video games can also scaffold players' metastrategic competence: understanding when and why different strategies should be deployed. As a game progresses, different strategies can be revealed to players, along with instruction on when it is appropriate to use them. Such metastrategic competence does not appear to routinely develop in the absence of instruction. Kuhn and her colleagues have incorporated the use of specific practice opportunities and prompts to help children develop these types of competencies. For example, Kuhn et al. (2000) incorporated performance-level practice and metastrategic-level practice for sixth- to eighth-grade students. Performance-level exercise consisted of standard exploration of the task environment, whereas metastrategic-level practice consisted of scenarios in which two individuals disagreed about the effect of a particular feature in a multivariable situation. Although no performance differences were found between the two types of practice with respect to the number of valid inferences, there were more sizeable differences in measures of metastrategic understanding. Similarly, Zohar and Peled (2008) examined the benefits of focusing on metastrategic competence during instruction of the control-of-variables strategy (CVS). Fifth-graders were given a metastrategic knowledge intervention in which CVS was described, and the circumstances under which it should be used were discussed. The intervention led to both strategic and metastrategic gains. It is clear from these studies that although meta-level competencies may not develop routinely, they can certainly be learned via explicit instruction.

Given the developmental trajectories of metacognitive, metamemory, and metastrategic skills, one could argue that video games cannot support the development of scientific reasoning skills in learners who lack the necessary metacognitive competences to take advantage of such support. Alternatively, it may be the case that video games scaffold developing metacognitive skills and make it possible for children to develop their scientific reasoning skills at a greater rate. Use of cognitive and metacognitive strategies is context-dependent (Cheng and Annetta, 2012). Ceci and Bronfenbrenner (1985) found an effect of context for children's behavior on two prospective memory tasks: taking cupcakes out of the oven and disconnecting charging cables from a motorcycle battery. Children performed the tasks more efficiently, and were better able to engage in concurrent tasks, in their own homes than in a university laboratory. In a similar vein, when given tasks concerning the specific knowledge domains of chess and football, children's performance depended on their domain-specific knowledge rather than their intelligence (Chi, 1978; Schneider et al., 1989). Because video games provide familiar contexts for many children, it could certainly be the case that games facilitate the use of metacognitive strategies compared to more formal educational or laboratory contexts. Cheng and Annetta (2012) provide evidence in favor of the argument that a video game context can promote metacognitive strategies. They used a serious educational game (“serious” in this context refers to a deliberate effort to educate) to instruct middle-school students about the effects of methamphetamine on the brain. Qualitative analyses of students' answers to post-task interview questions revealed that students monitored their learning, evaluated the information presented, showed awareness of the new knowledge they were acquiring, and made judgments of learning.

## Discussion and conclusions

In this paper we have attempted to outline a rationale for why we believe that video games have the potential to be exploited for educational gain. We argue that science, and science education, are highly social, collaborative, and scaffolded activities that are culturally situated and depend upon the use of cultural tools. We suggest that video games should be considered a type of cultural tool that can be used to scaffold science learning. Specifically, we proposed that video games have the potential to facilitate learning both science content and science process skills. Further, we suggested that elements of video games (e.g., they are intrinsically motivating and often make use of elements of science process) could be used to improve classroom-based science education. Although there are many other mechanisms by which games can be thought to afford learning, we focused our analysis on viewing video games as motivational scaffolds, cognitive scaffolds, and metacognitive scaffolds.

### What's the evidence?

As mentioned previously, there is evidence that video games affect general cognitive domains, such as spatial cognition and probabilistic inferences (e.g., Feng et al., 2007; Green et al., 2010; Bavelier et al., 2012). However, there is not yet much evidence for the effectiveness of games in science instruction or learning (O'Neil et al., 2005; Ke, 2009). The impact of games has been limited to particular components of education, for example, Malone (1981) and Malone and Lepper (1987) report that games create highly motivated learning conditions. There is little evidence that games, on their own, promote developing scientific skills, understanding of science content, or an understanding of the nature of science.

Recent reviews of research on gaming in education have all supported the same conclusion: right now, there is inconclusive and insufficient evidence for us to make any strong claims about the efficacy of video games. The National Research Council (2011) convened a panel of experts to investigate the academic potential of video games and computer simulations for learning science. Their analysis of video games focused on games that support inquiry approaches to science education. For example, computer simulations were found to have a greater influence on science learning, relative to non-science games. The conclusion of the National Research Council (2011) report was that the current state of research on the use of video games to support learning in science is “inconclusive.” Nevertheless, the report includes an entire chapter focused on future research aimed at exploring the “great potential” that games and simulations have for science learning. Concurrently, Tobias and Fletcher (2011) synthesized research on games designed for learning contexts. Although not specific to science, the overall conclusion of their volume is consistent with that of the National Research Council (2011) report. They also suggest that additional research is necessary to demonstrate the effectiveness of games in educational contexts. Young et al. (2012) also reviewed existing research on video games and academic achievement in math, science, language learning, history, and physical education. Their conclusion was that “many educationally interesting games exist, yet evidence for their impact on student achievement is slim” (p. 61). Similarly, although their review did support the effectiveness of educational games, they recommend the need for more research. A review of gaming in education by McClarty et al. (2012) also outlines the many theoretical reasons why games *should* be effective (e.g., forced mastery, motivation, feedback, engagement, planning, fun failure, choice, agency). However, they also come to the conclusion that although games like *Crystal Island*, *River City*, and *Quest Atlantis* incorporate what is known about cognition and motivation, research supporting their effectiveness is still inconclusive. They reiterate that games may work best when coupled with other pedagogy, and “will not replace teachers and classrooms, but they might replace some textbooks and laboratories” (p. 13).

### Next steps

When two review articles and two book-length reviews all conclude that there is insufficient or inconclusive evidence to support the idea that video games are effective at promoting learning in educational contexts, is it rational for researchers to continue to devote time and resources to this line of inquiry? Although these findings could be interpreted in a pessimistic light, Zimmerman (in press) argues that we should continue to conduct research on whether elements of video game play can be exploited for educational gain, and outlines a new research agenda. First, video games used to scaffold skills in science education must be of high quality. Second, we need precise research questions. Although previous reviews point to the common conclusion that research to date is “inconclusive” it may be that research thus far has been guilty of proposing overly broad research questions. Third, we need to identify the problems in science education that need solutions. These include specific problems that we also identified at the beginning of our article, such as recruiting and retaining people in STEM careers, and general problems such as promoting scientific literacy in the wider population. Fourth, in the light of identified problems, we need to specify high-level, and specific, learning goals. Fifth, as we are interested in science education, we need to consult the psychological research on scientific thinking when designing educational curricula that incorporate video games as a means of developing scientific skills. Sixth, we need to map the specific learning goals that have been identified to learning activities and measurable outcomes. Seventh, we need to conduct high-quality research on the effects of particular video games on specific science education outcomes. Finally, we need to see games as cultural tools for science education, rather than potential replacements for science instruction.

### Problems with educational games

Squire (2008) notes a number of ways in which educational games are different from commercial games. Serious games are often not as sophisticated as commercial games. The Research and Development that is available for commercial games is typically much more complete than for educational games. Commercial games are developed, programmed, and tested using teams of experts with access to substantial capital, while those with content expertise and little game expertise often create educational games. The time on task that is allowed for educational games is also quite different than a commercial game. For example, a user may spend 20 h per week on a game for many months in the quest to master it, whereas the typical science lesson is much shorter. It is also likely that serious games may be more likely to mimic typical classroom instruction, rather than harnessing the engaging elements that are built into commercial games. Habgood and Ainsworth (2011) note that while commercial games are intrinsically motivating, many educational games use gameplay as a reward for learning educational content, thus the gameplay becomes an extrinsic motivator. They argue that educational games should feature *intrinsic integration*, wherein the learning activities are tightly integrated with the gameplay and occur during the most engaging parts of a game, thus exploiting the flow generated by the game. Furthermore, the game should be used as a context in which to present external representations of the concepts to be learned.

Another potential difficulty is the transfer of learning from the gaming context to a novel (e.g., non-gaming) context. Children often demonstrate knowledge within the context in which it was learned, yet fail to transfer this knowledge even to closely related contexts (Klahr and Chen, 2011; Schwartz et al., 2011). Scientific skills cannot be assumed to transfer even within sub-disciplines of the same scientific domain. For example, Schunn and Anderson (1999) found differences between cognitive psychologists and social/developmental psychologists on a task in which the participants were asked to design an experiment to test two theories of human memory. One consistent finding is that transfer is more likely given high similarity between the context in which learning occurred and the target context (Klahr and Chen, 2011). Gaming contexts may be even less likely to promote transfer because they are often highly dissimilar from traditional educational contexts (e.g., lab experiments in science classes).

A promising example of an educational game that embodies many of the principles discussed in this paper is *Operation ARA* (Butler et al., 2011; Halpern et al., 2012). This game is designed to teach scientific reasoning and critical thinking skills to undergraduate students and advanced high schoolers. The key concept of identifying flawed scientific research is embedded in the gameplay and made more interesting by situating the problem in a context of an alien conspiracy to trick humans. *Operation ARA* is built on pedagogical principles, such as spaced practice and adaptive tutoring, and incorporates the motivational tools of interactivity, feedback, and incentives. Preliminary results indicate that students who played *Operation ARA* demonstrated more knowledge of scientific research methods than a control group.

### Conclusions

Rather than think of modern science education as “broken,” we prefer to consider the ways in which we have learned enough basic research about motivational, cognitive, and metacognitive development to engineer a superior product (i.e., learning experience). We suggested that games are cultural and educational tools for science education and that games have unique strengths that can be used to augment science education. Incorporating games into science instruction requires careful consideration of their strengths (e.g., intrinsically motivating) and weaknesses (e.g., unclear links to science content). In order to achieve maximum benefit, like any tool games need to be used at the right time in the right way. Rather than thinking of video games as the next potential educational panacea, we need to reframe the question/goal. We return to our analogy of promoting optimal health. Optimal instruction, like optimal health, is the result of many contributing factors, rather than a single, causal factor. Although each contributes, none on their own guarantees optimal health in the absence of the others. There is no “magic bullet” related to optimal health of health and there is no “magic bullet” in education. As in health, knowledge of the constituent components that contribute to effective education allows us to create contexts in which student learning is more likely. One of the components that we feel has the potential to contribute to modern education is the “gamification” of particular elements of science education.

### Conflict of interest statement

The authors declare that the research was conducted in the absence of any commercial or financial relationships that could be construed as a potential conflict of interest.
